# Ultra-high field MR angiography in human migraine models: a 3.0 T/7.0 T comparison study

**DOI:** 10.1186/s10194-019-0996-x

**Published:** 2019-05-06

**Authors:** Casper Emil Christensen, Samaira Younis, Ulrich Lindberg, Vincent Oltman Boer, Patrick de Koning, Esben Thade Petersen, Olaf Bjarne Paulson, Henrik Bo Wiberg Larsson, Faisal Mohammad Amin, Messoud Ashina

**Affiliations:** 1 Danish Headache Center and Department of Neurology, Rigshospitalet Glostrup, Faculty of Health and Medical Sciences, University of Copenhagen, Valdemar Hansens Vej 5, 2600 Glostrup, Denmark; 2 Functional Imaging Unit, Department of Clinical Physiology, Nuclear Medicine and PET, Rigshospitalet, Faculty of Health and Medical Sciences, University of Copenhagen, Copenhagen, Denmark; 3 Danish Research Centre for Magnetic Resonance, Centre for Functional and Diagnostic Imaging and Research, Amager and Hvidovre Hospital, Copenhagen, Denmark; 4 Division of Image Processing, Department of Radiology, Leiden University Medical Center, Leiden, Netherlands; 5 Center for Magnetic Resonance, Department of Health Technology, Technical University of Denmark, Kgs Lyngby, Denmark; 6 Neurobiology Research Unit, Department of Neurology, Rigshospitalet Blegdamsvej, Faculty of Health and Medical Sciences, University of Copenhagen, Copenhagen, Denmark

**Keywords:** Middle meningeal artery, Dura mater, Neurovascular, Sildenafil, Calcitonin gene-related peptide

## Abstract

**Background:**

Sildenafil and calcitonin gene-related peptide both dilate the intradural segments of the middle meningeal artery measured with 3.0 tesla (T) MR angiography. Here we hypothesized that an increase in field strength to 7.0 T and concomitant enhanced voxel resolution would lower variance in measurements of dilation in the intradural middle meningeal artery.

**Methods:**

Five subjects completed two sessions at respectively 3.0 T *and* 7.0 T. Each session comprised MR angiography scans once before and twice after administration of sildenafil, calcitonin gene-related peptide or placebo in a three-way, crossover, double-blind, placebo-controlled design.

**Results:**

Standard deviations of arterial circumference revealed no difference between 3.0 T and 7.0 T measurements (*p* = 0.379). We found a decrease in standard deviation from our original angiography analysis software (QMra) to a newer (LAVA) software package (*p* < 0.001). Furthermore, we found that the dilation after sildenafil and calcitonin gene-related peptide were comparable between 3.0 T and 7.0 T.

**Conclusions:**

Our findings suggest no gain from the increase in voxel resolution but cemented dilatory findings from earlier. The implemented software update improved variance in circumference measurements in the intradural middle meningeal artery, which should be exploited in future studies.

**Trial registration:**

The study is part of a parent study, which is registered at ClinicalTrials.gov (NCT03143465).

## Background

Neurovascular mechanisms of migraine headache has been studied extensively using magnetic resonance angiography (MRA) [1–5]. The middle meningeal artery (MMA) is an essential focus of these studies, both in search for the origin of pain and as a physiological measure of altered neurovascular modulation [6]. Two MRA studies found the extracranial part of MMA to be dilated more on the pain side than non-pain side in migraine patients during provoked attacks in human migraine models [1, 4]. If MMA dilation reflects activation of trigeminal nociceptors and meningeal neuropeptide release, then the *intracranial* segments of MMA might represent more local changes in the dural environment than the extracranial part.

In an effort to study dilation in the intracranial segments of the MMA, we recently performed a 3.0 tesla (T) MRA study in healthy volunteers showing dilation of the intradural MMA after administration of headache-inducing substances calcitonin gene-related peptide (CGRP) and sildenafil [7]. Changes in MMA dimensions within subjects over time in previous MRA studies vary from ~ 30% [3] to as little as ~ 5% [4] which challenges detection rate and power in costly and time-consuming MRI studies. Furthermore, the route of the MMA in the cranial convexity potentially limits the cross-sectional MRA resolution [8].

In this study, we wanted to investigate whether the uncertainty – defined as standard deviation (SD) of measurements along the course of the intradural MMA – improves when increasing the field strength from 3.0 T to 7.0 T, potentially increasing spatial resolution and diminishing the partial volume effect. We used a randomized, placebo-controlled, three-way, crossover, double-dummy setup at 7.0 T in participants who had previously gone through the exact same setup at 3.0 T and we compared standard deviations in measurements between the two systems.

## Methods

### Participants and approvals

We recruited healthy volunteers through a Danish recruitment website (www.forsoegsperson.dk). Participants were offered enrollment in this 7.0 T continuation study upon completion of the prior 3.0 T study, results of which have been published elsewhere [7]. Both men and women were eligible for inclusion if they were between 18 and 50 years of age, weighed 50 to 100 kg and had successfully completed the 3.0 T part of the parent study. Exclusion criteria were a history of somatic and/or psychiatric disease any primary headache disorder, other than infrequent episodes of tension type headache (less than 2 days/month), having first-degree relatives with migraine, being pregnant or breastfeeding, use of daily medication, other than oral contraceptives and not using safe contraceptive methods. Furthermore, volunteers were excluded if there were contraindications for MRI scans (i.e. ferromagnetic implants, recent surgical procedures, claustrophobia, etc.) or any other condition deemed by the investigator to be incompatible with participation. Participants provided written informed consent in accordance with the declaration of Helsinki and the study was approved by the Ethics committee of the Capital Region of Denmark (H-15019063) along with the Danish Medicines Agency (CIV-16-12-017964). The study is part of a parent study, which is registered at ClinicalTrials.gov (NCT03143465) and other parts of the study have been or will be published elsewhere.

### Study design

Participants were allocated to receive CGRP, sildenafil and placebo in random order on three separate study days. Drug administration was preceded by a 7.0 T MRA and succeeded by two additional scans at 30 min and 120 min post-administration. On each day, participants would receive an i.v. infusion of isotonic saline or CGRP as well as an oral administration of 100 mg sildenafil or placebo, where the combination was dependent on randomization (placebo+CGRP/sildenafil+placebo/placebo+placebo). This design was a replica of the previous 3.0 T study which all participants had previously completed at Rigshospitalet Glostrup, resulting in paired observations between 3.0 T and 7.0 T.

### Experimental procedures

Participants arrived at the Danish Research Centre for Magnetic Resonance headache-free on the morning of each study day. They were required to avoid intake of any medication (other than oral contraceptives), coffee, tea, cocoa, other caffeinated beverages and methylxanthine-containing food 12 h before the first scan and to fast 4 h before commencing the experiment. After arrival, a peripheral venous catheter was placed in a cubital vein and participants were placed in the scanner. They were instructed to stay awake during the recordings.

MR angiography scans were obtained at baseline (T_Baseline_) followed by drug administration at T_0_ and two additional MRI scans at T_30_ and T_120_. Participants were monitored with an Expression In Vivo monitor (Philips Medical, Orlando, FL, USA) continuously, regarding blood pressure, heart rate, end-tidal CO_2_, respiratory rate and pulse oximetry every 10 min following drug administration. A standardized headache questionnaire was used to evaluate any headache-related symptoms for the duration of the study where pain was scored on a numerical rating scale (NRS) from 0 (no pain) to 10 (maximum imaginable pain).

### Data acquisition

The acquisition of the 3.0 T scans used for comparisons in this study are described in detail elsewhere [7]. The 3.0 T MRI scans were performed on a Philips Achieva dStream Scanner (Philips Medical Systems, Netherlands) using a 32-channel phased-array head coil. A three-dimensional time-of-flight (TOF) MRA was conducted with the following parameters:

Field of view 200 × 200 × 36.75 mm^3^, acquired matrix size 800 × 570, acquired voxel resolution 0.25 × 0.35 × 0.70 mm^3^, reconstructed voxel resolution 0.20 × 0.20 × 0.35 mm^3^, TR 23 milliseconds, TE 3.5 milliseconds, flip angle 18°, SENSE p reduction 2.5, 3 chunks, duration 13 min 06 s.

The 7.0 T MRI scans were performed on a 7.0 T Philips Achieva Scanner (Philips Medical Systems, Netherlands) using a two-channel volume transmit head coil with 32-channel receiver array (Nova Medical, Inc., Burlington, MA, US). Again, a three-dimensional TOF MRA sequence was used with the following parameters:

Field of view 200 × 190.4 × 32.4 mm^3^, acquired matrix size 668 × 636, acquired voxel resolution 0.3 × 0.3 × 0.3 mm^3^, reconstructed voxel resolution 0.15 × 0.15 × 0.15 mm^3^, TR 19 milliseconds, TE 2.6 milliseconds, flip angle 30°, SENSE p reduction 4.5, 3 chunks, duration 9 min 58 s.

High-dielectric permittivity pads were placed over the temporomandibular joint to improve image quality at the base of the skull, where intradural segments of the MMA is located [9].

### Data analysis

MR angiography data was transferred to a separate work station in the DICOM format and analyzed using LKEB QMra, and the updated LKEB LAVA vessel contour detection software [10, 11]. The software allows the user to pinpoint start- and endpoint of vessels for contour analysis, propagates a center line through the vessel and provides circumference slices perpendicular to this line along the chosen segment. If slices were distorted, noisy or otherwise immeasurable, they were discarded. The user made sure to select the same segments within each subject between scans to provide reliable measurements of change over time. We analyzed the intradural segment of MMA, identified as the innermost part covered by the field of view, approximately 20 mm after MMA enters the skull through the foramen spinosum. Ultimately, a 5 mm segment is chosen comprising up to 34 slices and the mean and standard deviation is computed for that segment at that time point. 7.0 T MRA resolution required us to use updated contour detection software (LAVA), which was not used in the original study, causing us to reanalyze the previous data using the new software post hoc. To further explore the potential effect of the resolution on 7.0 T, we resampled the 7.0 T data, decreasing the resolution, to mimic the original 3.0 T scans. Both QMra and LAVA analyses are reported, and the software change was factored into analyses (see below). All analyses of 3.0 T and 7.0 T scans were performed on the same workstation by CEC.

### Statistical analyses

Our primary endpoint was difference in SDs of vessel segment measurements in intradural MMA at 3.0 T vs. 7.0 T within subject, within drug, within time point providing us with a maximum of 5_subjects_ x 3_days_ x 3_times_ x 2_sides_ = 90 paired observations. Vessel segment standard deviation was analyzed with a linear mixed effects model with scanner and contour detection software as fixed effects and subject, drug and time point as random effects. Secondary analyses on drug effect on circumference over time were performed descriptively as percentage change from baseline and using Bland-Altman agreement statistics between scanners [12].

All analyses were performed using R (version 3.4.2) with packages lme4 (version 1.1–17) and BlandAltmannLeh (version 0.3.1), *p*-values are reported as two-tailed with a significance level of 5%.

## Results

Five participants who had completed the 3.0 T parent study went on to complete the 7.0 T extension. Two men and three women mean age 24 (range 20 to 29). The median number of days between 3.0 T and 7.0 T studies were 476 (range 330 to 501).

**Fig. 1:**
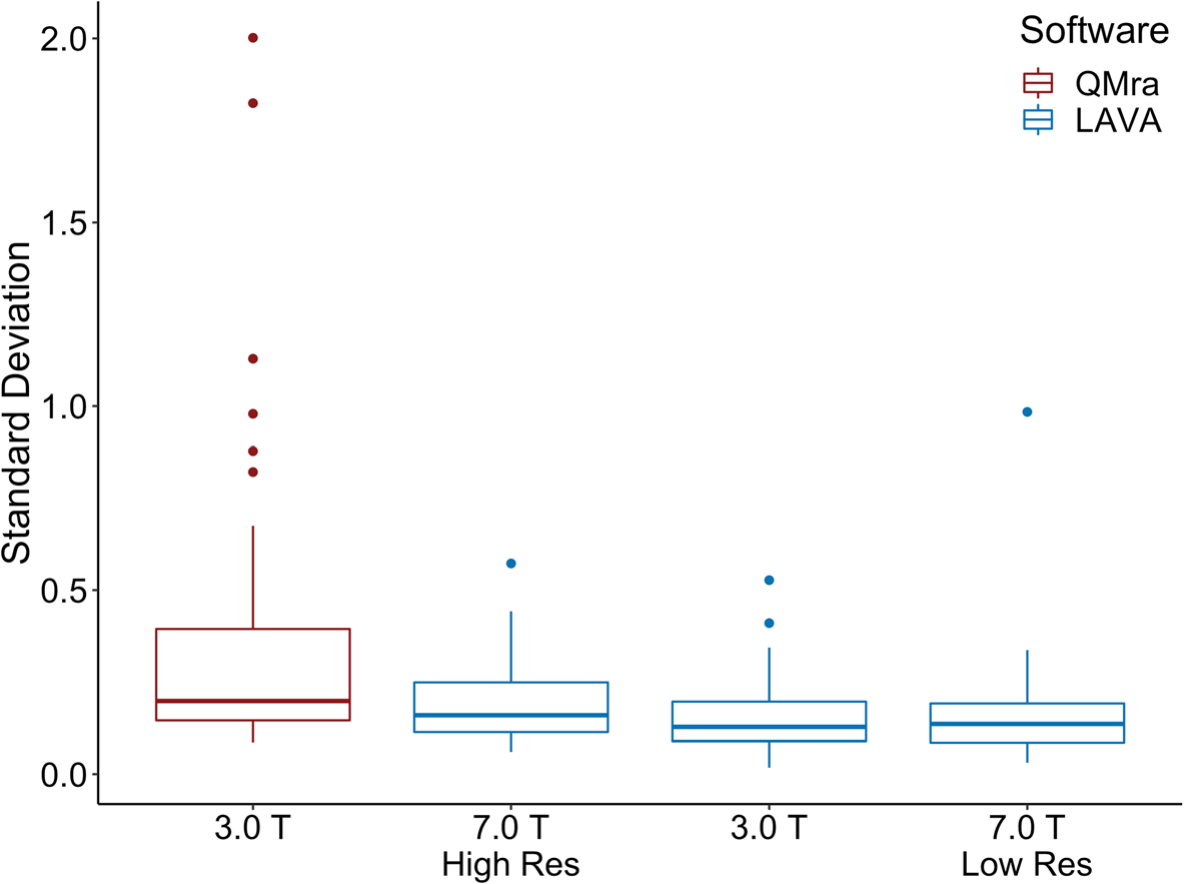
Standard deviations of the intradural MMA measurements in the four analysis iterations. 7.0 T High Res: 7.0 T scan at full resolution; 7.0 T Low Res: 7.0 T scan resampled to 3.0 T resolution

Mean SD for intradural MMA on 3.0 T with QMra software was 0.332 mm (SE 0.033), mean circumference was 4.18 mm (95% CI [4.04 to 4.32]). We found no difference in SD between 3.0 T and 7.0 T when applying the same LAVA software on both (*p* = 0.379), but SD was 0.184 mm lower for LAVA than for QMra independent of scanner field strength (*p* < 0.001). We found no difference between resampled and original 7.0 T standard deviations (Fig. 1).

**Fig. 2:**
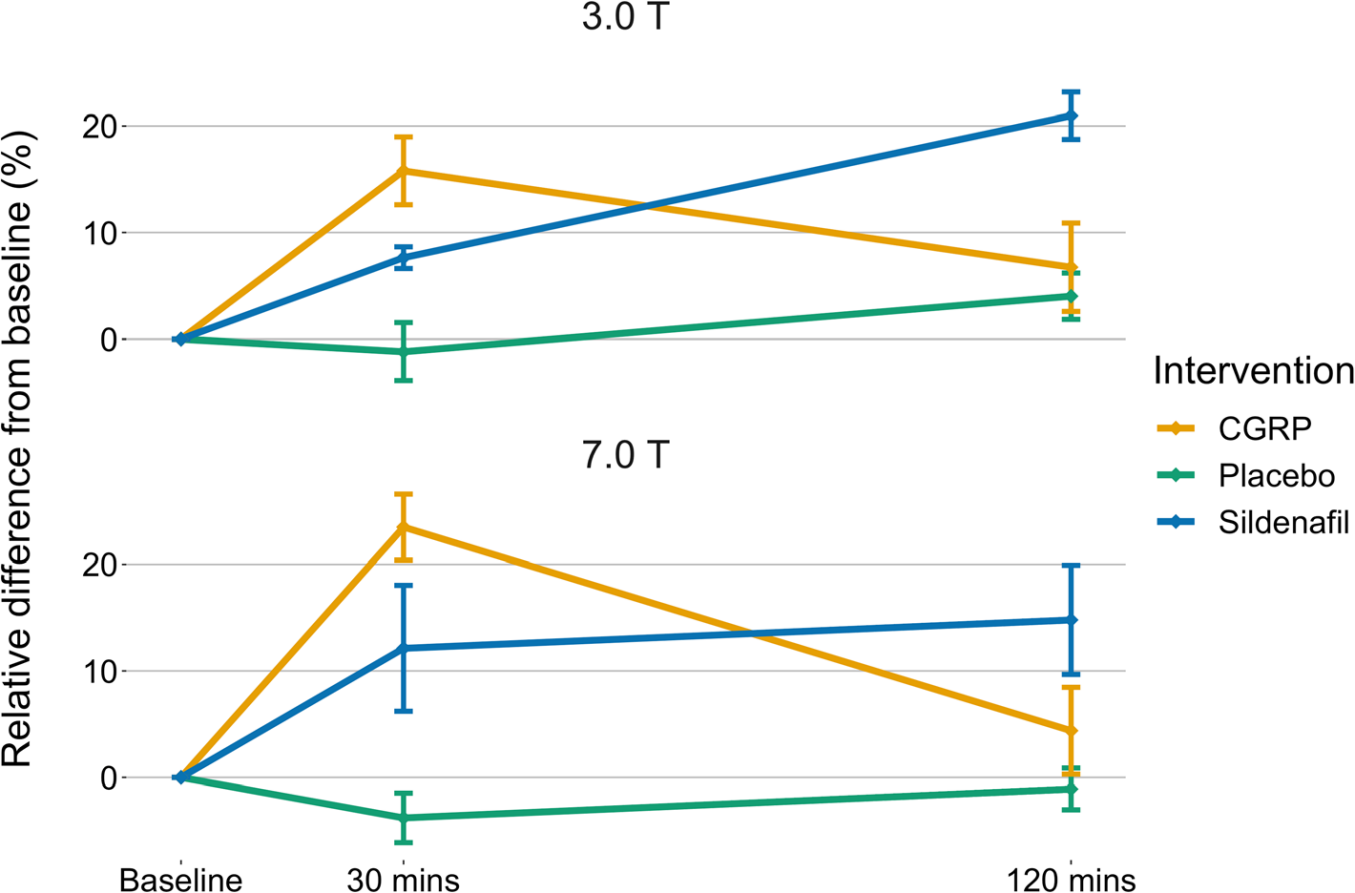
Change in intradural MMA circumference over time after each of the three interventions. 3.0 T and 7.0 T data is depicted as mean ± SE, all data analyzed with LAVA software

**Fig. 3:**
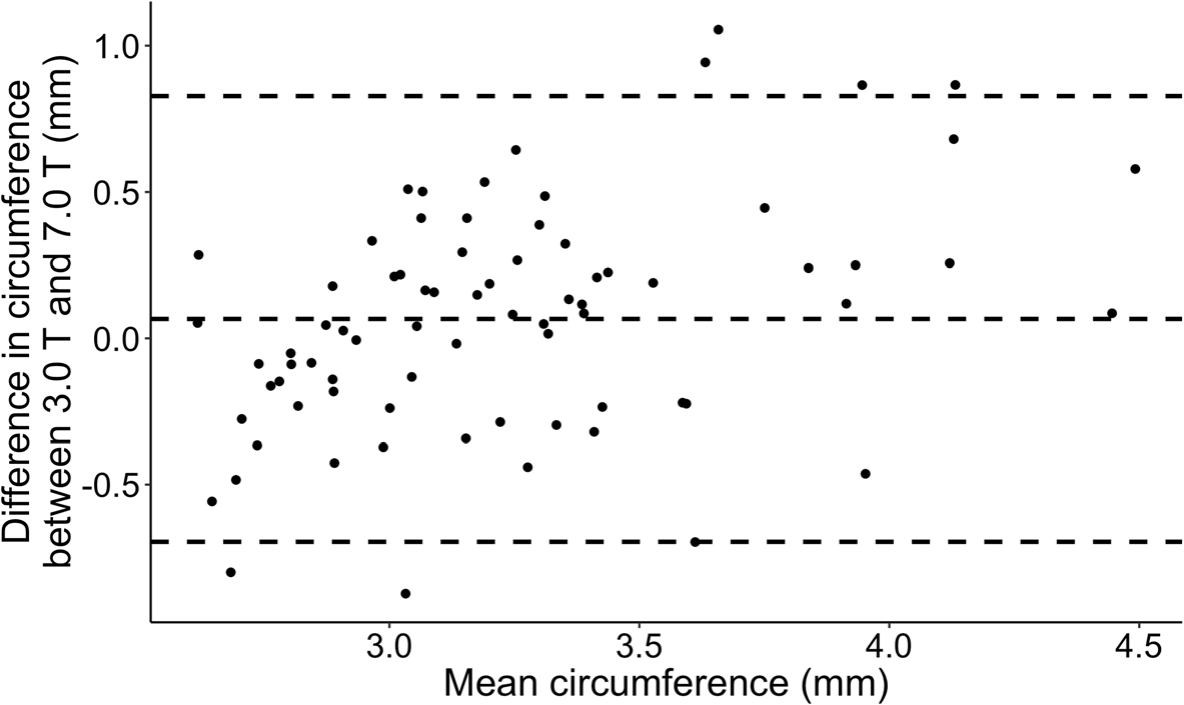
Bland-Altmann plot depicting agreement between circumference measurements of intradural MMA 3.0 T vs 7.0 T within subject, within drug, within time point analyzed with LAVA software. Each point represents difference in circumference between 3.0 T and 7.0 T as a function of mean of the two measurements. Dashed lines are overall mean of differences ±2SD

Arterial circumference change over time between the two scanners are shown in Fig. 2. We found similar patterns across the three interventions with immediate increase in arterial circumference both after CGRP (15.8% at 3.0 T and 23.5% at 7.0 T) and sildenafil (7.6% at 3.0 T and 12.1% at 7.0 T). Dilation after sildenafil appears to be sustained after 120 min (21.0% at 3.0 T and 14.8% at 7.0 T) contrary to the CGRP-induced dilation (Fig. 2). Correspondingly, Fig. 3 shows relatively high levels of agreement between circumference measurements at 3.0 T and 7.0 T within subject, drug and time (Fig. 3).

**Fig. 4:**
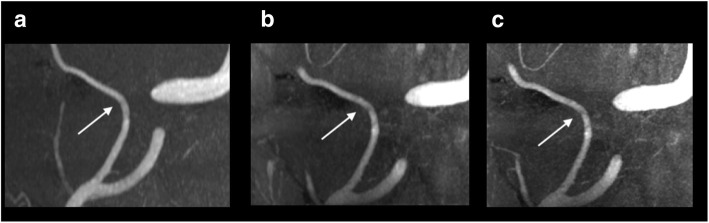
Examples of the middle meningeal artery (arrow) in three different data sets. **a**: 3.0 T scan; **b**: 7.0 T scan at low resolution; **c**: 7.0 T scan at high resolution

**Fig. 5:**
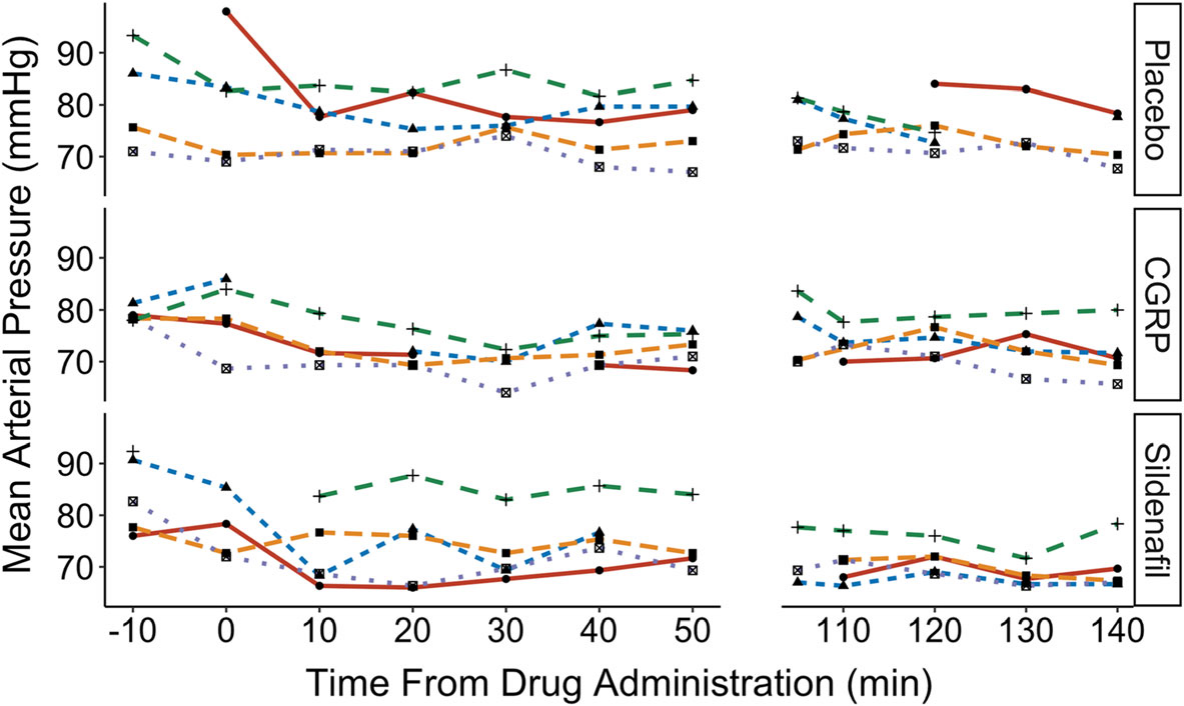
Mean arterial blood pressure by time in the individual participants after each of the three interventions

Examples of the different angiography recordings are shown in Fig. 4. We observed an apparent decrease in mean arterial pressure after sildenafil (Fig. 5) and a small increase in heart rate after CGRP (not shown).

## Discussion

The main outcome of this study was that we found no difference in standard deviation of measurements of intradural MMA circumference between 3.0 T and 7.0 T. We did, however, uncover a difference in standard deviation between the previously used QMra circumference detection software and the newer LAVA. Furthermore, we showed high robustness in measurements of vascular effects of CGRP and sildenafil over time as the temporal evolution from drug administration was similar between the 3.0 and 7.0 T scan days (Fig. 2).

Sildenafil is a selective phosphodiesterase-5 inhibitor that impedes degradation of cyclic guanosine monophosphate (cGMP) which ultimately leads to relaxation of vascular smooth muscle cells. [13] Calcitonin gene-related peptide relaxes vascular smooth muscle via a G-protein coupled receptor-mediated increase in cyclic adenosine monophosphate (cAMP) [14]. We have previously shown the ability to measure intradural MMA circumference increase after either sildenafil *or* CGRP using high resolution 3.0 T MRA [7]. The intradural branches of the MMA move in all three planes and we hypothesized, that we could improve confidence in our caliber measurements, if we streamlined all dimensions of the voxel size on MRA. In our previous study, the lowest cross-sectional resolution was 0.35 × 0.70 mm^2^, whereas at 7.0 T, we could increase the resolution to 0.3 × 0.3 mm^2^. However, this increase in resolution did not change the standard deviations along the vessel segment measurements. Neither did the temporal evolution of intradural MMA caliber change, when moving to 7.0 T, showing absolute robustness of our measurements from 3.0 T and consolidating our previous findings (Figs. 2 and 3).

The contour detection software works by detecting a pathline of the vessel chosen by the operator. Then a model is fitted to the underlying image data defining a 3D mesh in non-uniform rational basis spline fashion [11]. QMra software moves along the pathline and determines the best candidate in neighboring voxels as next in line in the vessel contour [10]. The newer LAVA program scans a wider range of potential voxels and locates the best candidates using the maximum gradient [11]. The older QMra software is thus more susceptible to poor pathline estimations and noise whereas LAVA looks at a broader range of voxels and is more resilient to these effects.

In MRA research in migraine, changes in dimensions of the MMA have been proposed a marker of meningeal activation during migraine attacks, and changes reported are as small as ~ 5% corresponding to around 0.15 mm in the intradural MMA circumference [4]. Needless to say, a high level of confidence in measurements is needed, and here we show robust MMA measurements from our 3.0 T results even when re-testing at ultra-high field strength.

### Strengths and limitations

We tested standard deviations along vessel segments in randomized, double-blind, placebo-controlled fashion using the same subjects on the two different systems resulting in quite strong paired comparisons. Furthermore, contour detection is a semi-automated procedure with minimal operator influence [15]. A central limitation is the time between 3.0 T and 7.0 T scans with a median of 476 days between them.

## Conclusions

We found no reduction in standard deviation with the increase in voxel resolution from 3.0 T to 7.0 T. The implemented software update, however, improved variance in circumference measurements in the intradural MMA, which should be exploited in future studies. Furthermore, we affirmed the dilatory response in intradural MMA after sildenafil and CGRP that was reported previously [7].

## Abbreviations

cAMP: cyclic Adenosine Monophosphate
cGMP: cyclic Guanosine Monophosphate
CGRP: Calcitonin Gene-Related Peptide
MMA: Middle Meningeal Artery
MRA: Magnetic Resonance Angiography
NRS: Numeric Rating Scale
SD: Standard Deviation
T: Tesla
TOF: Time-Of-Flight
