# Correction to: Ultra-high field MR angiography in human migraine models: a 3.0 T/7.0 T comparison study

**DOI:** 10.1186/s10194-019-1014-z

**Published:** 2019-05-28

**Authors:** Casper Emil Christensen, Samaira Younis, Ulrich Lindberg, Vincent Oltman Boer, Patrick de Koning, Esben Thade Petersen, Olaf Bjarne Paulson, Henrik Bo Wiberg Larsson, Faisal Mohammad Amin, Messoud Ashina

**Affiliations:** 1 Danish Headache Center and Department of Neurology, Rigshospitalet Glostrup, Faculty of Health and Medical Sciences, University of Copenhagen, Valdemar Hansens Vej 5, 2600 Glostrup, Denmark; 2 Functional Imaging Unit, Department of Clinical Physiology, Nuclear Medicine and PET, Rigshospitalet, Faculty of Health and Medical Sciences, University of Copenhagen, Copenhagen, Denmark; 3 Danish Research Centre for Magnetic Resonance, Centre for Functional and Diagnostic Imaging and Research, Amager and Hvidovre Hospital, Copenhagen, Denmark; 4 Division of Image Processing, Department of Radiology, Leiden University Medical Center, Leiden, Netherlands; 5 Center for Magnetic Resonance, Department of Health Technology, Technical University of Denmark, Kgs Lyngby, Denmark; 6 Neurobiology Research Unit, Department of Neurology, Rigshospitalet Blegdamsvej, Faculty of Health and Medical Sciences, University of Copenhagen, Copenhagen, Denmark

**Correction to: J Headache Pain**

**https://doi.org/10.1186/s10194-019-0996-x**

After publication of the original article [1], the authors have notified us that an updated version of Figures 1, 2 and 3 should have been published. The incorrect and revised figures can be found below.

Incorrect figures: 
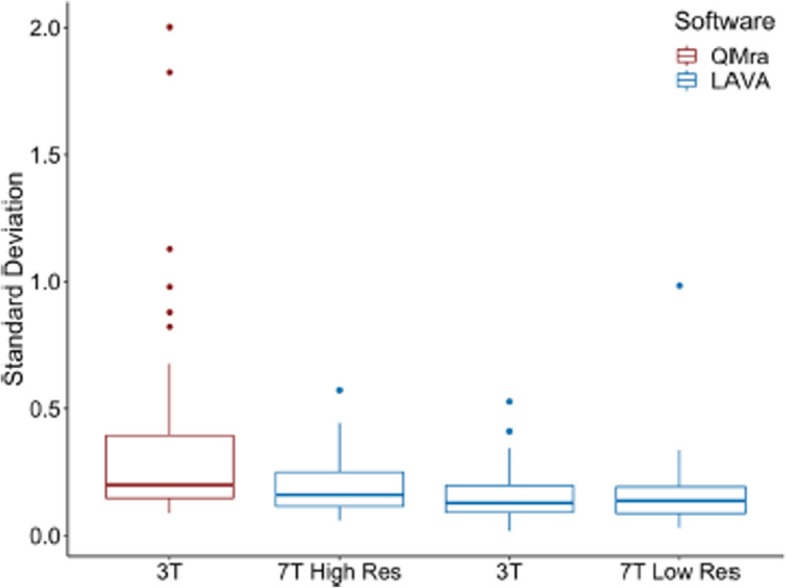

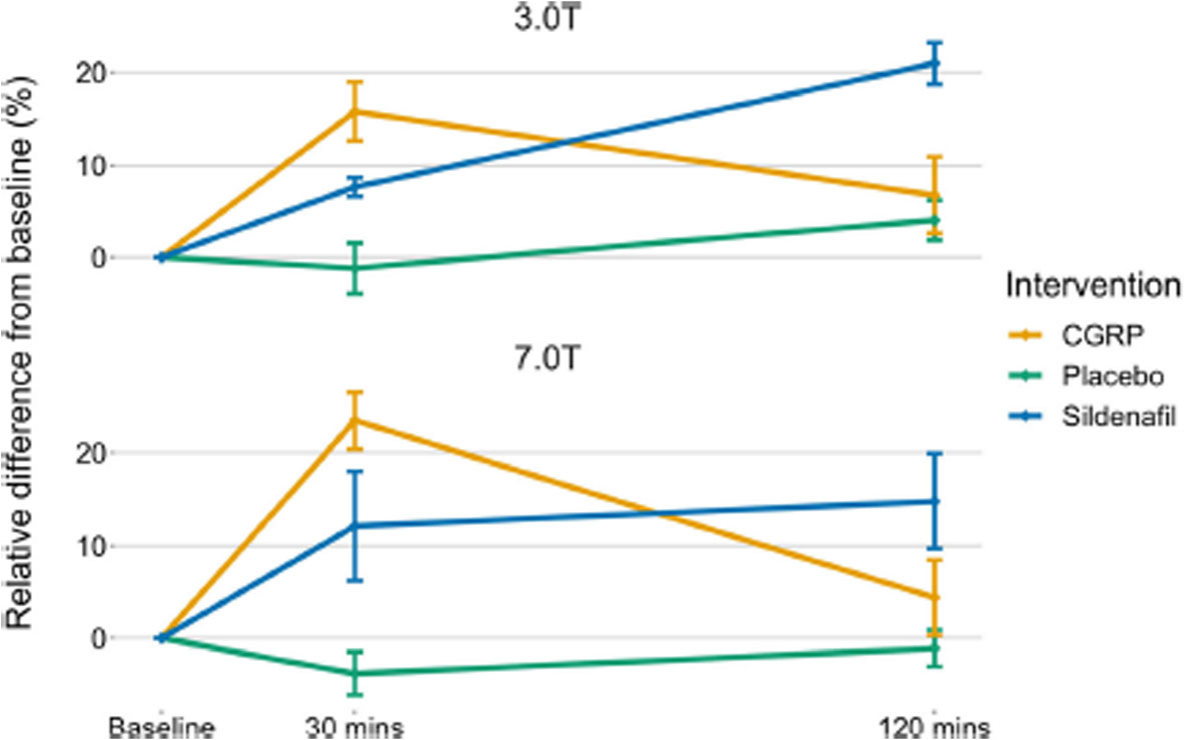

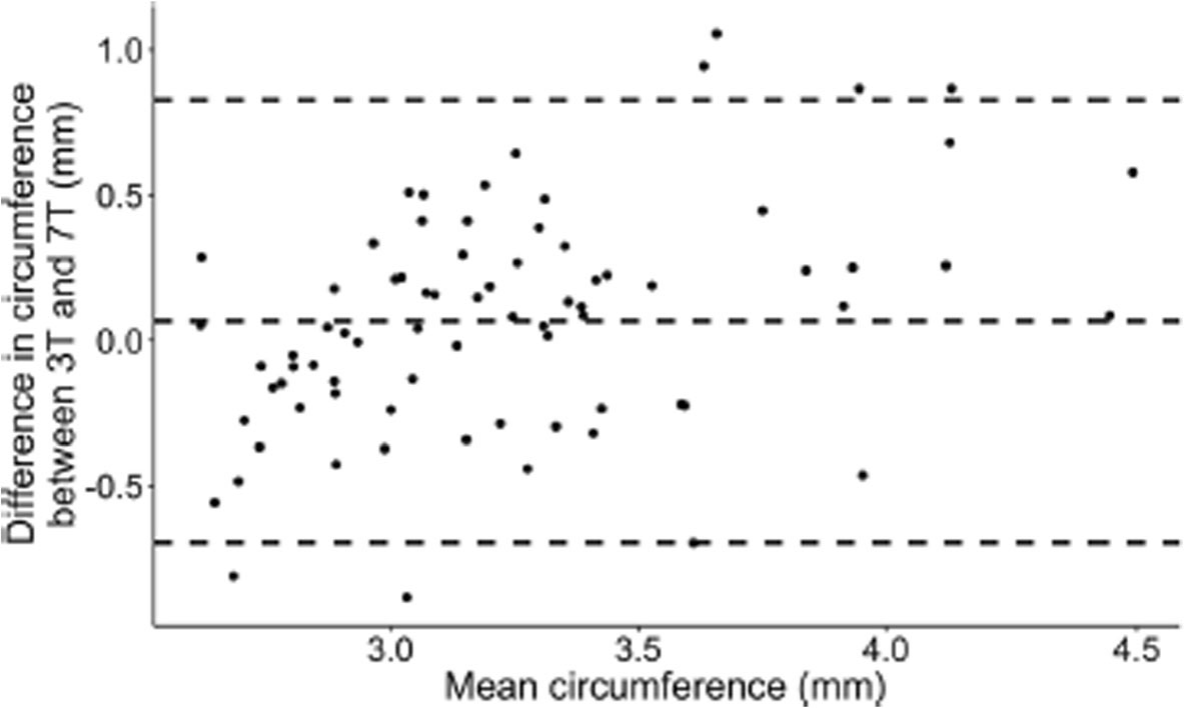


Revised figures:

**Fig. 1:**
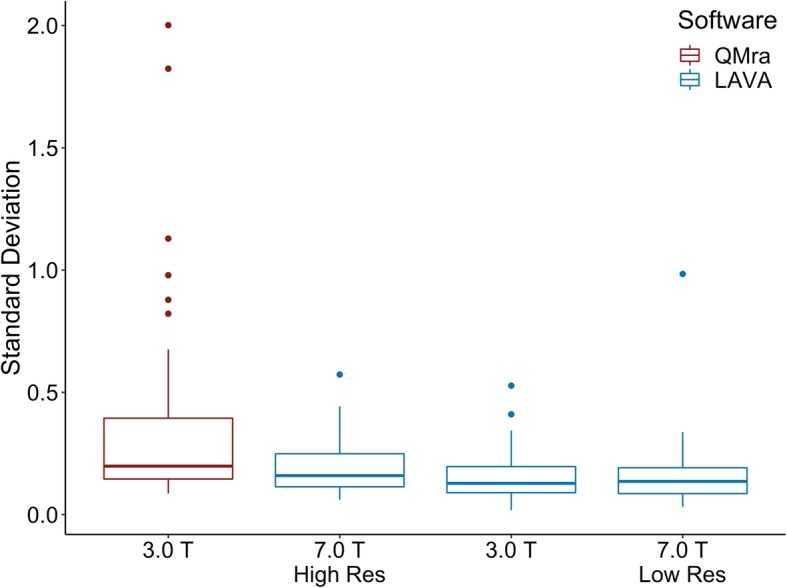
Standard deviations of the intradural MMA measurements in the four analysis iterations. 7.0 T High Res: 7.0 T scan at full resolution; 7.0 T Low Res: 7.0 T scan resampled to 3.0 T resolution

**Fig. 2:**
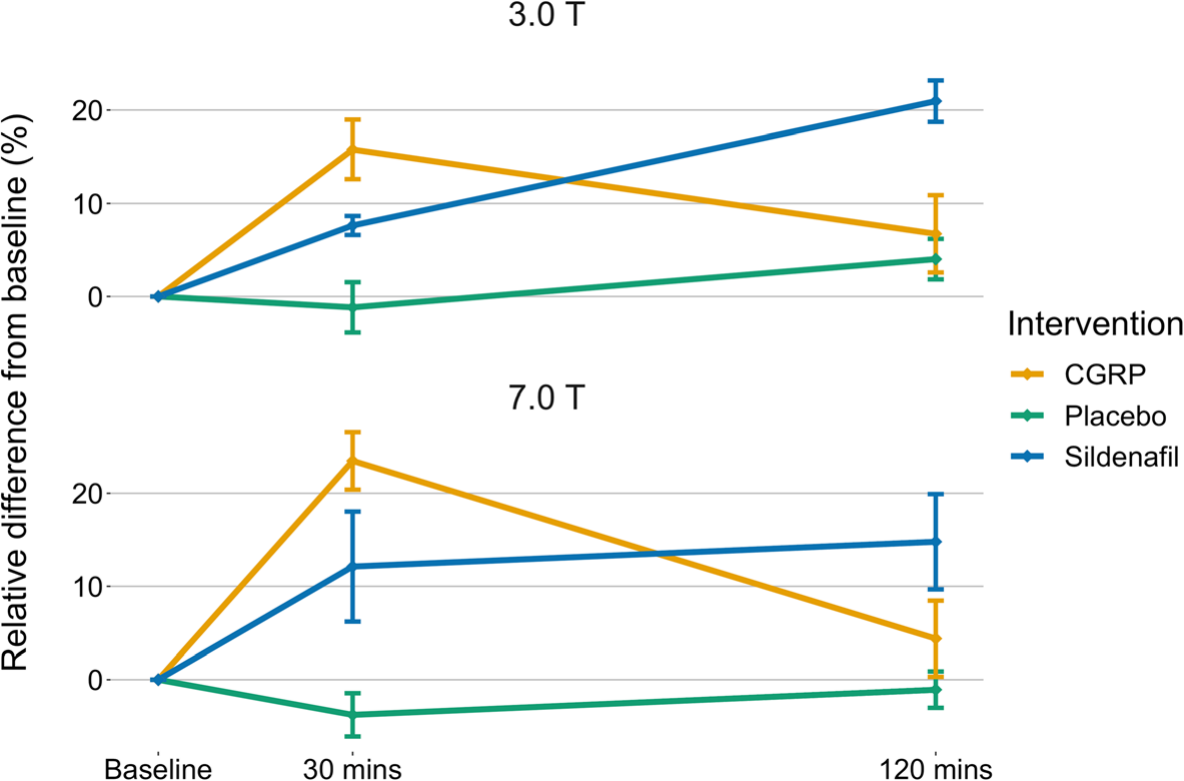
Change in intradural MMA circumference over time after each of the three interventions. 3.0 T and 7.0 T data is depicted as mean ± SE, all data analyzed with LAVA software

**Fig. 3:**
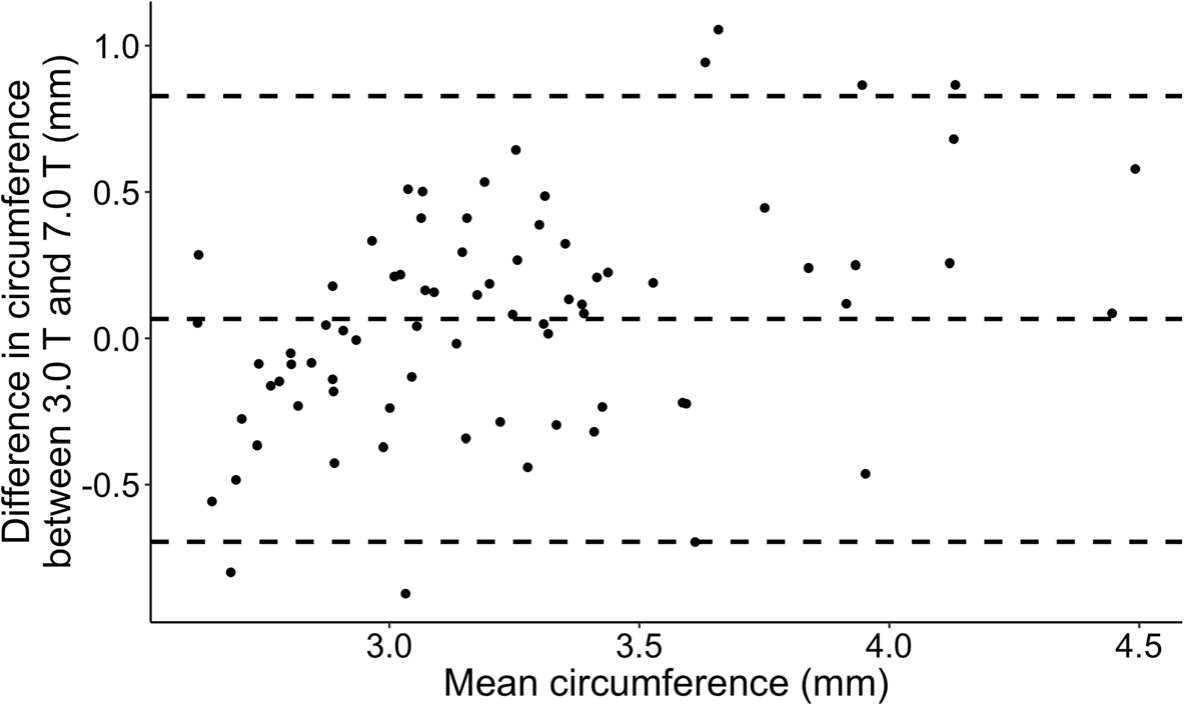
Bland-Altmann plot depicting agreement between circumference measurements of intradural MMA 3.0 T vs 7.0 T within subject, within drug, within time point analyzed with LAVA software. Each point represents difference in circumference between 3.0 T and 7.0 T as a function of mean of the two measurements. Dashed lines are overall mean of differences ±2SD

The original article has been corrected.

## References

[1] Christensen (2019). Ultra-high field MR angiography in human migraine models: a 3.0 T/7.0 T comparison study. J Headache Pain.

